# Salivary Biomarkers for Dental Caries Detection and Personalized Monitoring

**DOI:** 10.3390/jpm11030235

**Published:** 2021-03-23

**Authors:** Pune N. Paqué, Christopher Herz, Daniel B. Wiedemeier, Konstantinos Mitsakakis, Thomas Attin, Kai Bao, Georgios N. Belibasakis, John P. Hays, Joël S. Jenzer, Wendy E. Kaman, Michal Karpíšek, Philipp Körner, Johannes R. Peham, Patrick R. Schmidlin, Thomas Thurnheer, Florian J. Wegehaupt, Nagihan Bostanci

**Affiliations:** 1 Clinic of Conservative and Preventive Dentistry, Center of Dental Medicine, University of Zurich, Plattenstrasse 11, 8032 Zurich, Switzerland; thomas.attin@zzm.uzh.ch (T.A.); joel.jenzer@icloud.com (J.S.J.); philipp.koerner@zzm.uzh.ch (P.K.); patrick.schmidlin@zzm.uzh.ch (P.R.S.); thomas.thurnheer@zzm.uzh.ch (T.T.); florian.wegehaupt@zzm.uzh.ch (F.J.W.); 2 Austrian Institute of Technology, Molecular Diagnostics, Giefinggasse 4, 1210 Wien, Austria; christopher_herz@pall.com (C.H.); johannes.peham@ait.ac.at (J.R.P.); 3 Statistical Services, Center of Dental Medicine, University of Zurich, Plattenstrasse 11, 8032 Zurich, Switzerland; daniel.wiedemeier@zzm.uzh.ch; 4 Hahn-Schickard, Georges-Koehler-Allee 103, 79110 Freiburg, Germany; konstantinos.mitsakakis@hahn-schickard.de; 5 Laboratory for MEMS Applications, IMTEK-Department of Microsystems Engineering, University of Freiburg, Georges-Koehler-Allee 103, 79110 Freiburg, Germany; 6 Department of Dental Medicine, Division of Oral Diseases, Karolinska Institutet, 141 04 Huddinge, Sweden; kai.bao@ki.se (K.B.); george.belibasakis@ki.se (G.N.B.); nagihan.bostanci@ki.se (N.B.); 7 Department of Medical Microbiology and Infectious Diseases, Erasmus University Medical Centre Rotterdam (Erasmus MC), 3015 GD Rotterdam, The Netherlands; j.hays@erasmusmc.nl (J.P.H.); w.e.kaman@acta.nl (W.E.K.); 8 Academic Centre for Dentistry Amsterdam (ACTA), Department of Oral Biochemistry, Free University of Amsterdam and University of Amsterdam, 1081 LA Amsterdam, The Netherlands; 9 BioVendor-Laboratorní Medicína, a.s., Research and Diagnostic Products Division, Immunoassays, Clinical Validation & BioVendor Analytical Testing Service, Karasek 1767/1, 62100 Brno, Czech Republic; karpisek@biovendor.com

**Keywords:** diagnostics, interleukins, screening, personalized monitoring, saliva, biomarkers, caries, JAK, STAT

## Abstract

This study investigated the potential of salivary bacterial and protein markers for evaluating the disease status in healthy individuals or patients with gingivitis or caries. Saliva samples from caries- and gingivitis-free individuals (*n* = 18), patients with gingivitis (*n* = 17), or patients with deep caries lesions (*n* = 38) were collected and analyzed for 44 candidate biomarkers (cytokines, chemokines, growth factors, matrix metalloproteinases, a metallopeptidase inhibitor, proteolytic enzymes, and selected oral bacteria). The resulting data were subjected to principal component analysis and used as a training set for random forest (RF) modeling. This computational analysis revealed four biomarkers (IL-4, IL-13, IL-2-RA, and eotaxin/CCL11) to be of high importance for the correct depiction of caries in 37 of 38 patients. The RF model was then used to classify 10 subjects (five caries-/gingivitis-free and five with caries), who were followed over a period of six months. The results were compared to the clinical assessments of dental specialists, revealing a high correlation between the RF prediction and the clinical classification. Due to the superior sensitivity of the RF model, there was a divergence in the prediction of two caries and four caries-/gingivitis-free subjects. These findings suggest IL-4, IL-13, IL-2-RA, and eotaxin/CCL11 as potential salivary biomarkers for identifying noninvasive caries. Furthermore, we suggest a potential association between JAK/STAT signaling and dental caries onset and progression.

## 1. Introduction

The primary goal in dental medicine is the maintenance of oral health and the prevention of oral diseases, such as caries or periodontitis [1]. Those diseases are based on oral biofilms, which can develop on all the surfaces within the oral cavity [2,3]. A shift in the composition of the oral microbiome paves the way for opportunistic pathogens to induce disease outbreak and progression [4,5,6]. The development of caries lesions is driven by microorganisms, dietary habits (the frequency of carbohydrate intake, pH and stickiness of food debris), and host factors (salivary flow rates, immune responses, genetic predispositions [7] and hygiene measures) [8,9,10]. Microorganisms produce strong organic acids during their carbohydrate metabolism and induce mineral loss in the tooth substance leading to tooth decay [11]. Progressing caries lesions cause an inflammation of the pulp, which leads to pulpitis and periapical periodontitis. Some bacteria, such as *Streptococci* and *Lactobacilli*, are particularly associated with the development of these caries lesions [2,12]. Persisting biofilms around the teeth on the gingival sulcus can also induce gingivitis [13,14]. This periodontal disease induces inflammatory responses in the host, causing the marginal swelling of the gingiva, an elevated exudation of gingival crevicular fluid (GCF) and a successive increase in local pocket depths [15]. Gingivitis- and periodontitis-inducing biofilms are mainly associated with Gram-negative, anaerobic bacteria, while caries-associated bacteria are predominantly related with Gram-positive carbohydrate-fermenting bacteria [15]. However, the composition of the oral or tooth-specific microbiome may not be exclusively associated with the maintenance of oral health or disease [16]. It has been shown that patients who abstain from oral hygiene procedures subsequently develop different responses to their accumulated plaque [13,17,18]. These different clinical responses enabled a classification into “periodontal-resistant” and “periodontal-insufficient” or ”high responder” and “low responder”, respectively [18]. Hence, the host’s response to pathogenic stimuli also accounts for the maintenance of oral health or development of disease. As such, minor, pathogenic changes within the oral cavity can be diagnosed at an early stage, by the molecular analysis of host immune markers. Shifts in the biomarker composition within the GCF have been previously described in gingivitis patients [19,20,21]. For caries patients, the close proximity of odontoblasts and the dental pulp to the lesions causes host immune responses that lead to the production and release of several cytokines, chemokines, and growth factors [22]. Both the gingivitis and the caries inflammatory responses take place in the presence of saliva throughout the oral cavity. Thereby, this oral fluid acts as a collection matrix for released immune defense molecules and microorganisms in both diseases.

Most studies currently focus on detecting potential biofluid targets for periodontitis patients by using analytical procedures for biofluids such as saliva or gingival crevicular fluid. The current consensus among dental experts is leaning towards combined and multitargeted strategies for the identification of high-/low-risk patients, via the assessment of responses to therapy and the prediction of periodontal stability. This may be achieved by validated combinations of host-derived salivary biomarkers and key pathogens [23,24,25,26]. Common biomarker panels include cytokines (such as the interleukins IL-6 and IL-1β), matrix metalloproteinases (MMP-8 and MMP-9), a metallopeptidase inhibitor (TIMP-1) and the presence of periodontal pathogens (e.g., *Porphyromonas gingivalis*, *Treponema denticola*, *Tanerella forsythia, Fusobacterium nucleatum*, *Campylobacter rectus*, and *Prevotella intermedia*) [27,28,29,30,31,32,33].

Knowledge of dental pulp immune response markers is usually acquired by the direct assessment of pulp tissue and, less commonly, by the analysis of pulpal blood, peripheral blood serum, GCF, dentinal fluid, or extracellular pulpal fluid [22,34]. Unfortunately, relatively little is known about the combination of salivary biomarkers required to identify caries patients or patients prone to caries [35,36]. In fact, only a few studies have indicated differences in the salivary protein composition between healthy and caries patients [37,38,39], with the salivary parameters investigated including basic proline-rich peptides [37], the total protein load [38], and the total protein composition by molecular weight pattern [39]. Molecular analyses for salivary caries signatures remain comparatively unexplored.

The direct contact of saliva with oral lesions, as well as saliva’s simple availability, highlights its potential use as a diagnostic medium for the detection of oral diseases [40,41]. Furthermore, due to the above-mentioned multifactorial etiology of caries and associated challenges in the development of prevention and treatment strategies, there is a need for rapid personalized diagnostics [42,43]. In fact, the discovery of suitable salivary biomarkers might enable the early identification of high-risk patients prone to developing gingivitis, whilst allowing the early detection of caries lesion development. Furthermore, such biomarkers could be used to assess the status of orally healthy individuals, including in patient monitoring, over time.

Therefore, the current study set out to analyze the saliva of 73 subjects, who were grouped based on clinical diagnosis into healthy individuals, patients with gingivitis and patients with untreated deep caries lesions. Central to the study was an exploratory multimethod approach aiming to identify suitable biomarkers that could predict dental caries. A secondary aim was to detect gingivitis patients within the same cohort. Additionally, 10 subjects (five caries-/gingivitis-free and five with caries) were followed over a period of six months, and saliva was collected for biomarker analysis. It was hypothesized that a combination of protein and microbial candidate markers could enable a differentiation between all groups and be further validated with the 10 follow-up subjects.

## 2. Materials and Methods

### 2.1. Study Population and Study Design

**Figure 1:**
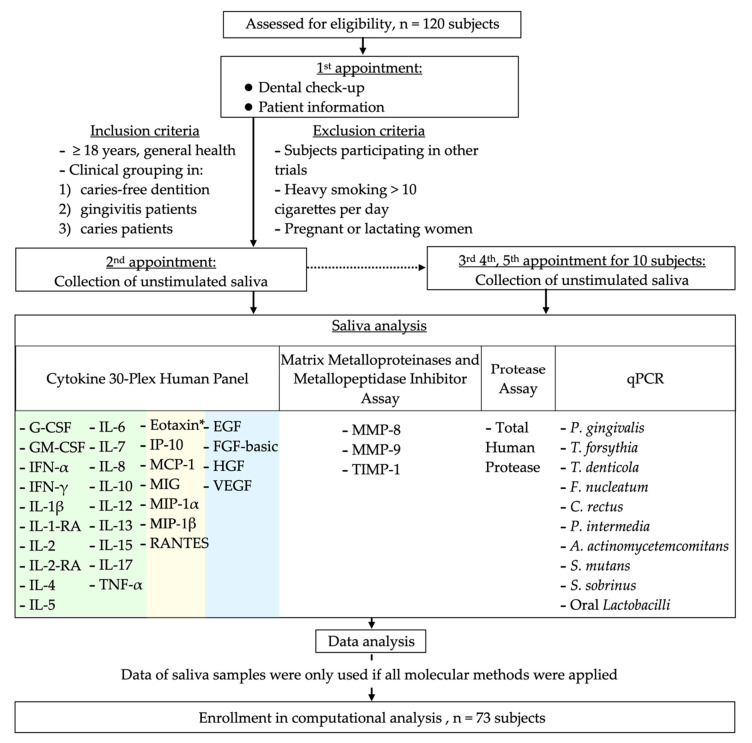
Flow-chart showing the study design, study population, and applied saliva analyses. Biomarkers of the Cytokine 30-Plex Human Panel are color-coded for cytokines (green), chemokines (yellow), and growth factors (blue). * Eotaxin/CCL11.

Individuals who came for a check-up or dental treatment to the Center of Dental Medicine, Zurich, and who fulfilled the inclusion/exclusion criteria were recruited for the study (approved by the local Swiss ethics committee with the BASEC-no. 2016-00435, date of approval: 09.01.2016). A total of 120 subjects (Figure 1) were asked to participate in a previous study (minimum age of 18 years; mean, 35.8 years; 57 females) and donate saliva for a microbial analysis and identification of diseases with a qPCR (quantitative polymerase chain reaction) assay [12]. Out of this cohort, stored (−80 °C, not longer than six months) saliva was used from healthy subjects (*n* = 29; mean age, 31.8; 18 females), patients with gingivitis (*n* = 22; mean age, 35.4; 13 females), and patients with open caries lesions (*n* = 69; mean age, 37.6; 26 females). Complete data for all the applied methods (44 biomarkers) were available for 73 of the initially collected 120 samples and were included in the computational prediction analysis: healthy patients (*n* = 18), patients with gingivitis (*n* = 17), and open caries lesions (*n* = 38); see Section 2.7. The participating subjects visited the Center of Dental Medicine twice. The first appointment (30–60 min) included a dental examination by the treating dentist. Eligible subjects who signed written informed consent visited the Center for a second appointment (within two weeks), during which saliva collection took place.

The general health status and potential in- and exclusion criteria of the subjects were assessed by a dentist during the first visit. Systemically healthy subjects were asked to participate in this study with a selected oral health status (general health was indicated by the exclusion of patients with diabetes; heart disease; infections, such as tuberculosis, hepatitis, sexually transmitted diseases, and HIV/AIDS; tumor diseases; and gastric digestive disorders causing vomiting). Subjects were excluded if they had already participated in another clinical trial within the last three months prior to the collection of saliva, were heavy smokers taking > 10 cigarettes per day (e-cigarette usage was not separately surveyed), or were pregnant or lactating women. The clinical examination allowed a grouping of subjects into orally healthy (no caries lesions, and periodontal screening index (PSI) scores of 0 in ≥4 sextants and ≤2 in ≤2 sextants), gingivitis (no caries lesions, and PSI scores of 1 in ≥4 sextants and ≤2 in ≤2 sextants), or caries patients (≥2 open dentinal caries lesions and PSI scores of ≤2 in ≥ 6 sextants).

Patient enrollment and saliva collection took place from September 2018 to April 2019 and were approved by the local Swiss ethics committee (BASEC-no. 2016-00435). Each subject donated saliva once (baseline). For further investigations, 10 subjects (five caries-/gingivitis-free and five with caries) of the cohort were asked to repeat the saliva donation four, five, and six months after baseline. The donated saliva of each participating subject was anonymized during collection, and access to patient data was only available to authorized study personnel. All the saliva samples were screened for 44 candidate biomarkers (cytokines, chemokines, growth factors, matrix metalloproteinases, a metallopeptidase inhibitor, proteolytic enzymes, and selected oral bacteria). These biomarkers were selected as a multitargeted approach to addressing different immune responses, which were, in part, already described as correlating with oral diseases [27,28,29,30,31,32,33].

### 2.2. Saliva Sampling

#### 2.2.1. Baseline Collection of Saliva

As described in a previous study [12], a strict saliva donation protocol was followed. Briefly, the participants were asked not to eat, drink sugary drinks, or perform any oral hygiene measures the night before the saliva donation. However, water intake was permitted at all times. A standardized saliva donation was timed between 8.00 and 10.00 am [44,45] and performed to obtain unstimulated whole saliva [46]. A video was provided to instruct the subjects on the methodology for standardized saliva donation. A collection time of 15 min was scheduled, and the saliva was collected in a test tube. Tubes containing less than 1.8 mL of saliva were discarded. Tubes with sufficient volumes were then vortexed, aliquoted in DNA low-bind tubes or protein low-bind tubes (Eppendorf, Wesseling-Berzdorf, Germany) depending on the following assay, and stored at −80 °C.

#### 2.2.2. Repeated Measurements of Saliva in Selected Subjects

Ten representative subjects of the cohort (five caries-/gingivitis-free and five with caries) were followed over a period of six months (Figure 1). Analogous to timepoint 0 (baseline), the patients received three further oral checkups and were grouped into their respective clinical groups, i.e., “healthy”, “gingivitis”, or “caries”. During the following observation time, three successive saliva collections took place, at four, five, and six months after the baseline collection. The patients were asked at each saliva collection appointment to answer questions regarding the dental treatments performed, such as the treatment of caries lesions, the extraction of teeth, and whether a professional tooth cleaning had taken place. Additionally, the patients were asked about antibiotic intake, changes in their nutrition regimes, and their self-care plaque control. The oral health statuses of the patients were assessed during each saliva collection appointment by dental specialists. Saliva samples were collected following the same protocol as used for the baseline saliva collection, coded, and stored for further analysis of the 44 markers as previously described. The resulting dataset consisting of the repeated measurements of 10 additional individuals was later used as an independent dataset to check the statistical models and is therefore also referred to as the “test dataset”.

### 2.3. 30-Plex Analysis of Biomarkers in Saliva

For each sample, the aliquot with the highest volume was thawed from −80 °C overnight at 6 °C on an orbital shaker at 180 rpm to ensure homogeneity. It was then centrifuged at 21,000 rcf for 3 min at 4 °C. A total of 25 µL of the supernatant was used for the analysis with the Cytokine 30-Plex Human Panel (Cat. # LHC6003M, by Thermo Fisher Scientific, Supplementary Table S1). The manufacturer’s recommendations were followed throughout the analysis. Cytokine capturing was performed by incubating overnight. The biotinylated antibody step was extended to 1 h, and the R-phycoerythrin (R-PE) coupling, to 2 h. The instrumental setup was established according to the 30-Plex Human Panel protocol with a target bead count of 50. Data acquisition was carried out on a Luminex 200 system (Invitrogen, 4088 Commercial Ave, Northbrook, IL, USA) and its xPONENT software (Version 3.1, Invitrogen, Waltham, MA, USA) package. The plates were measured twice, on both low and high Photomultiplier Tube (PMT) settings.

### 2.4. MMP-8, MMP-9, and TIMP-1 Measurements

The MMP-8, MMP-9, and TIMP-1 concentrations were determined by using commercial ELISA kits (BioVendor-Laboratorní medicína a.s., Brno, Czech Republic), according to the manufacturer’s instructions [47,48]. The calibration range was adapted for the determination of MMP-8 in saliva samples. Saliva supernatant from each patient sample was centrifuged at 4 °C for 10 min at 10,000× *g* and then diluted 20, 40, or 250 times, respectively, with an appropriate dilution buffer, and used for analysis.

### 2.5. Protease Analysis

Total salivary proteolytic activity was measured as described previously [49]. In brief, 49 µL of saliva was incubated with 1 µL of 800 µM PEK-054 ([FITC]-NleKKKKVLPIQLNAATDK-[KDbc]), a substrate used to assess total protease activity. A trypsin solution from bovine pancreas (500 U, Sigma-Aldrich Chemie B.V., Zwijndrecht, The Netherlands) was used as a standard. The reactions were incubated at 37 °C for 60 min, and the increase in fluorescence was determined using a fluorescence microplate reader (FLUOstar Galaxy, BMG Laboratories) at an excitation wavelength of 485 nm and an emission wavelength of 530 nm. All the saliva samples were analyzed in duplicate.

### 2.6. qPCR

#### 2.6.1. DNA Extraction for qPCR

DNA was extracted using the GenElute^TM^ Bacterial Genomic DNA Kit (Sigma-Aldrich, St. Louis, MO, USA) and the protocol for Gram-positive bacterial preparation and Streptococcus species (with the addition of 250 units/mL mutanolysin) with a prolonged lysis step. For each patient, 920 µL of saliva was spun down at 18,000 rcf for 3 min. The remaining pellet was resuspended in an enzyme solution consisting of lysozyme and mutanolysin and incubated for 1 h at 37 °C on a Thermomixer (1400 rpm, Eppendorf, Wesseling-Berzdorf, Germany). RNase treatment was conducted according to the manufacturer’s recommendations. The incubation time during proteinase K treatment was prolonged to 30 min at 55 °C at 1400 rpm. The DNA was eluted in 135 µL of 10 mM TrisHCl (pH 8.8) and stored at −25 °C.

#### 2.6.2. Oligonucleotide Design and Primer and Probe Specificity

Novel primer and probe sets were designed for the bacterial *strains P. gingivalis, T. forsythia, T. denticola, F. nucleatum, C. rectus, P. intermedia, A. actinomycetemcomitans, S. mutans, S. sobrinus and orally associated Lactobacilli* [12]. Target sequences were extracted out of the “Nucleotide” database [50] provided by the National Center for Biotechnology Information (NCBI) and the “Human Oral Microbiome Database” (HOMD), courtesy of the Forsyth Institute in Cambridge, MA, USA [5]. The primer and probe sets targeted the 16S ribosomal RNA gene except for A. actinomycetemcomitans and S. sobrinus. The limited availability and heterogeneity of the A. actinomycetemcomitans 16S rRNA sequences in the NCBI Nucleotide database meant that the virulence factor and toxin gene LtxA was targeted instead. *Streptococci* display a high sequence similarity within their genera; hence, the 23S rRNA (uracil-5-)-methyltransferase RumA gene was selected to increase the specificity of detection. The oligonucleotide design was exclusively performed using Primer3 [51]. The oligonucleotide coverage, as well as specificity, was first confirmed in silico using Primer Blast in combination with the “not redundant/nucleotide (nr/nt)”, “chromosomes of all organism”, and “HOMD 16S rRNA RefSeq” databases. The results were validated in the lab using bacterial genomic reference DNA provided by the Leibniz Institute DSMZ, German Collection of Microorganisms and Cell Cultures GmbH (https://www.dsmz.de/), and the ATCC, American Type Culture Collection (www.lgcstandards-atcc.org) [12].

#### 2.6.3. Duplex qPCR

qPCR was performed using a dual-color format utilizing a custom “TaqMan Lyophilized 1-Step qPCR MasterMix” on a Roche Light Cycler 480II. Each reaction contained extracted patient material or reference genomic DNA and an internal amplification control, which consisted of 0.01 ng of genomic DNA of Serinicoccus marinus. Probes targeting orally associated bacteria were labelled with 6-carboxyfluorescein (6-FAM), whereas the internal control probe used Roche’s proprietary LightCycler Red 610. A two-step cycling protocol was applied, starting with an initial activation for 2 min at 95 °C followed by 40 cycles alternating between 95 °C for 3 s and 60 °C for 30 s. First, external standard curves were generated in three consecutive qPCR runs for each oral taxon using the bacterial genomic reference DNA highlighted in Section 2.6.2. The standards included six concentrations in 10-fold increments from 10 ng to 0.1 pg [12]. The values for each standard were then converted into genome equivalents by extracting the genome size of each strain from the NCBI Assembly [52], multiplying it by the average molecular weight of a base pair, and correlating it to the six standard concentrations. Subsequently, the extracted patient DNA was subjected to qPCR, and the corresponding Cq values were directly quantified and converted into genome equivalents on the basis of the previously established standard curves.

### 2.7. Statistics and Computational Analysis

The data of a saliva sample were only used if all the molecular methods were applied on that sample, resulting in the measurement of 44 markers in 73 samples. All the measurements are expressed as genome equivalents, absorbance units (AU), pg/mL, or ng/mL (depending on the biomarker) and compiled in a spreadsheet. Only data above the detection limit (depending on each biomarker) were used directly (Supplementary Table S2). Measurements below the detection limit were imputed using the half-minimum method [53].

The baseline data (“training dataset”) were used for principal component analysis (PCA) on the correlation matrix and for training the random forest (RF). Moreover, this dataset was also the basis for comparing the marker levels of IL-4, IL-13, IL-2-RA, and eotaxin/CCL11 between healthy individuals, patients with gingivitis, and patients with untreated deep caries lesions using nonparametric one-way ANOVAs (Kruskal–Wallis rank sum tests) followed by pairwise comparisons according to Conover. The additional data from recalling 10 subjects (“test dataset”) were prepared in the same way, but were only used to check RF predictions. RF was chosen over other algorithms because it offers powerful prediction properties in combination with fairly interpretable model characteristics and fits [54,55]. All the computational analyses were performed with the statistical software R [56], including the packages ggplot2 [57], PMCMRplus [58], randomForest [59], and FactoMineR [60].

## 3. Results

The saliva samples of 120 subjects were collected and analyzed with different molecular methods, screening for 19 cytokines, 7 chemokines, 4 growth factors, 2 metalloproteinases, 1 metallopeptidase inhibitor, 1 protease, and 10 orally associated bacteria (Figure 1). Data were only subjected to further statistical modeling if results for all the assays (44 prominent protein and DNA markers) were available. Altogether, this accounted for 73 saliva samples, of which 18 were grouped as healthy, 17 as gingivitis, and 38 as caries patients.

### 3.1. Frequency of Detected Biomarkers

**Figure 2:**
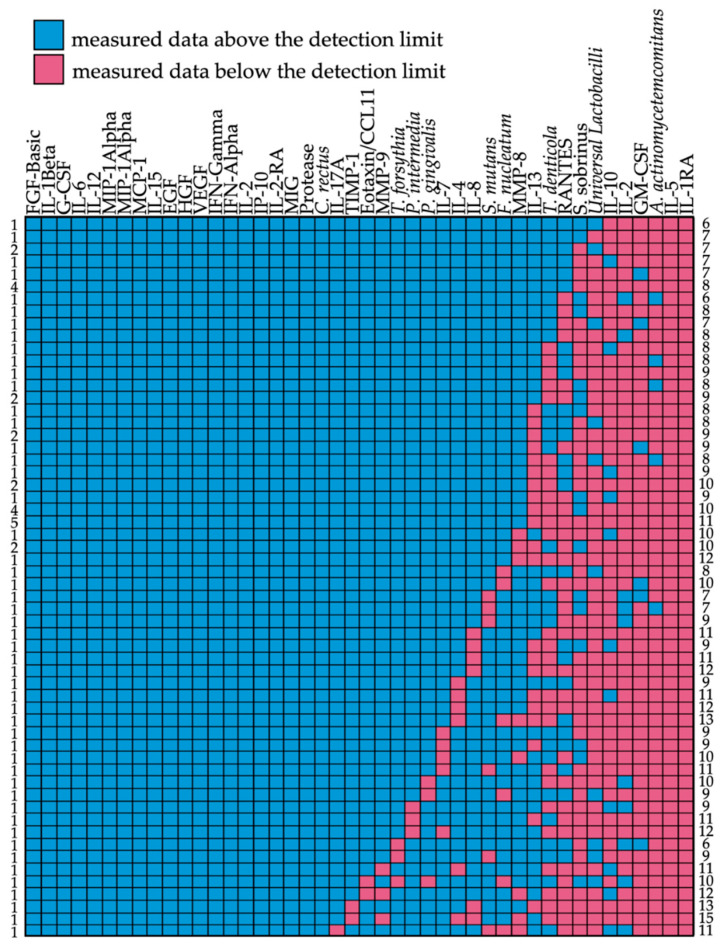
Detection matrix of all biomarkers (x-axis) in the analyzed 73 subjects (y-axis). Color codes show data measured above (blue) or below (red) the detection limit. Numbers on the left correspond to the numbers of patients with the same pattern of biomarkers above/below the detection limit. Numbers on the right represent the numbers of biomarkers below the detection limit per line.

Data were gathered at various time points by project partners throughout this study. An initial visualization enabled the assessment of data quality as shown in the detection limit matrix (Figure 2). It can be seen that data for A. actinomycetemcomitans, S. sobrinus, and oral Lactobacilli were below the detection limit in the majority of the subjects. Furthermore, there were no data for all the IL-1-RA and IL-5 measurements. The remaining assays, however, showed a very robust performance (data above the detection limit; see Supplementary Table S2). 

### 3.2. Principal Component Analysis (PCA)

**Figure 3:**
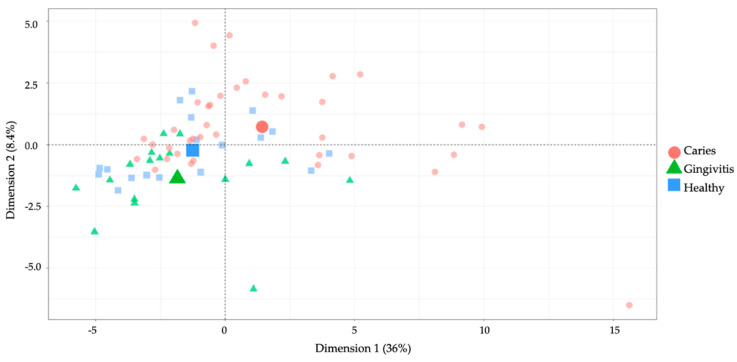
Results obtained from a principal component analysis (PCA) with the first dimension on the x-axis (36%) and the second dimension on the y-axis (8.4%). The groups are separated by color and shape (caries = red square; gingivitis = green triangle; caries = red circle). Three bigger shapes are located at the center of gravity for each group.

The first principal component encompassed 36% of the total variance in the data, while the second principal component absorbed 8.4% of the total variance (Figure 3). There was a profound overlap between the healthy and gingivitis groups, while the caries group could generally be better differentiated. This suggested that the groups exhibited distinctive marker profiles.

### 3.3. Biomarkers for Discriminating the Oral Health Status

**Figure 4:**
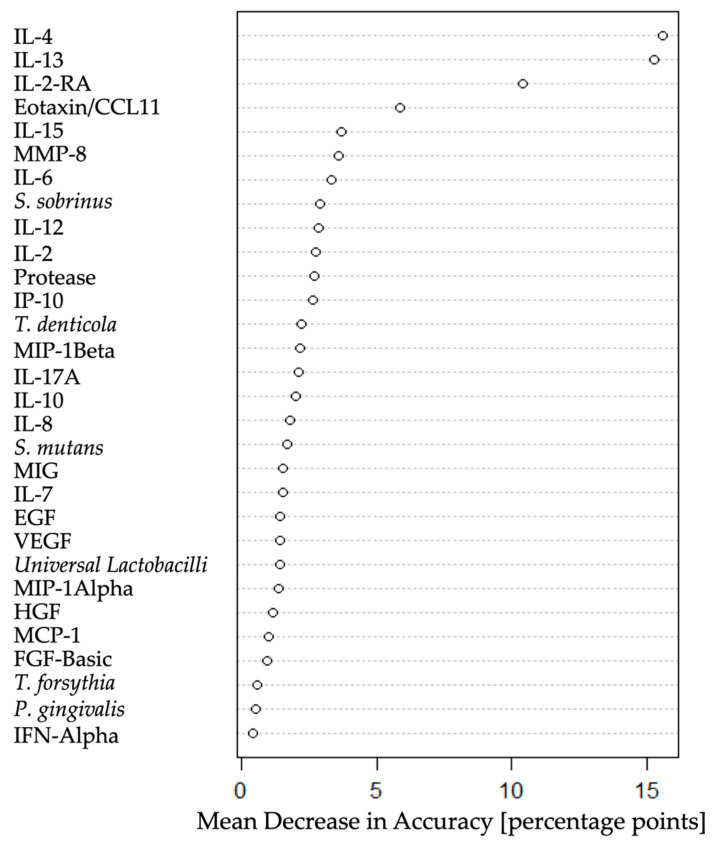
Random forest results plotted according to mean decrease in accuracy, show the strongest discriminators, namely, IL-4, IL-13, IL-2-RA, and eotaxin/CCL11.

Following the PCA, another multidimensional approach (a random forest supervised learning model) was applied to the training dataset in order to extract the strongest discriminators that were potentially capable of correctly assigning patients into the three clinical study groups. Figure 4 illustrates the strongest discriminators plotted according to the mean decrease in accuracy: IL-4, IL-13, and IL-2-RA, followed by eotaxin/CCL11. These were the four classifiers that enabled the best distinction of caries, healthy, and gingivitis patients considering all the measured markers.

**Figure 5:**
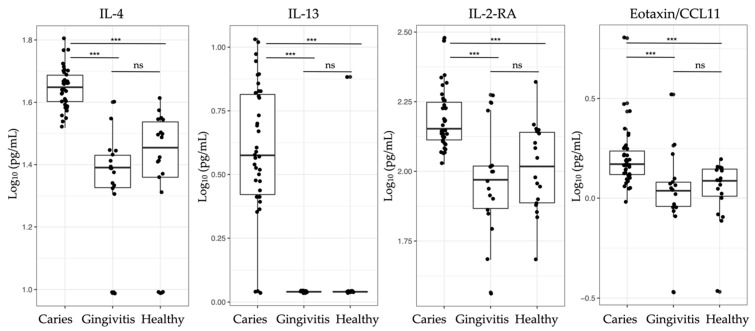
Boxplots of the four strongest group classifiers (IL-4, IL-13, IL-2-RA, and eotaxin/CCL11) based on random forest classification, with median values and interquartile ranges for each group shown (healthy individuals, patients with gingivitis, and those with caries). *p*-values were derived from Kruskal–Wallis tests followed by post hoc pairwise comparisons according to Conover. ns = *p* > 0.05; *** *p* ≤ 0.001 (see Table 1 for specific *p*-values).

Interestingly, only four markers were needed to generate the best discrimination between the different oral health groups, i.e., IL-4, IL-13, and IL-2-RA as well as the chemokine eotaxin/CCL11 (Figure 4). Oral bacteria did not play an important role. No statistically significant differences were found between healthy and gingivitis patients regarding the tested biomarkers (Table 1). Nevertheless, there was a clear statistically significant difference between the caries and the other two clinical groups (Figure 5, Table 1). Elevated biomarker levels could be observed for all four biomarkers in caries, when compared to those in the healthy and gingivitis groups. The mean data ± standard deviation was calculated for all the biomarkers. The biomarker levels of the patients with deep caries lesions were 44.9 pg/mL ± 6.8 pg/mL for IL-4, 4.6 pg/mL ± 2.6 pg/mL for IL-13, 158.9 pg/mL ± 44.4 pg/mL for IL-2-RA, and 1.7 pg/mL ± 0.9 pg/mL for eotaxin/CCL11. The biomarker IL-13 was below the detection limit in the healthy and gingivitis groups.

### 3.4. PCA Loading Plot

**Figure 6:**
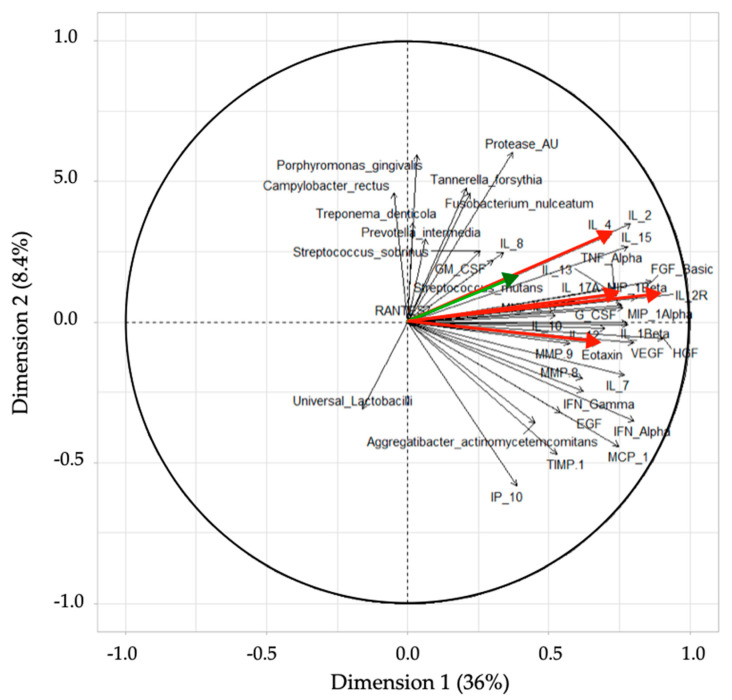
PCA loading plot visualizing the correlation between the biomarkers tested and clusters of samples, grouped based on their similarity. The red-colored arrows show the four biomarkers (ordered from top to bottom): IL-4, IL-13, IL-2-RA, and eotaxin/CCL11. The *S. mutans* arrow is colored in green, in close proximity to IL-4.

PCA loading plots were generated to visualize potential correlations between the tested biomarkers and clusters of samples. The directions and angles of the plots showed a positive correlation between IL-2-RA and IL-13, followed by IL-4 (Figure 6). Another interesting observation is that periodontitis and caries-associated bacteria are not likely to be correlated, although, as expected, bacterial protease activity appeared to correlate better with the periodontitis-associated strains. The only caries-associated strain showing a positive correlation with IL-4, IL-13, and IL-2-RA was *Streptococcus mutans* (green).

An objective of this study was to utilize the measured biomarkers in order to predict the clinical health statuses of patients (Table 1). Using the RF algorithm, the results were then compared to the initial clinical assessments of dental specialists. The overall out-of-bag error amounted to 30%. However, there was a pronounced contrast in classification error between the individual groups, as can be seen in Table 2. The caries group could be clearly differentiated from the gingivitis and the healthy groups (classification error of 2.6%), while the gingivitis and healthy groups could not be satisfactorily distinguished from each other on the basis of the biomarkers that were investigated (classification errors of 58.8% and 61.1%, respectively).

### 3.5. Comparison of the RF Model Predictions and Clinical Assessments

**Figure 7:**
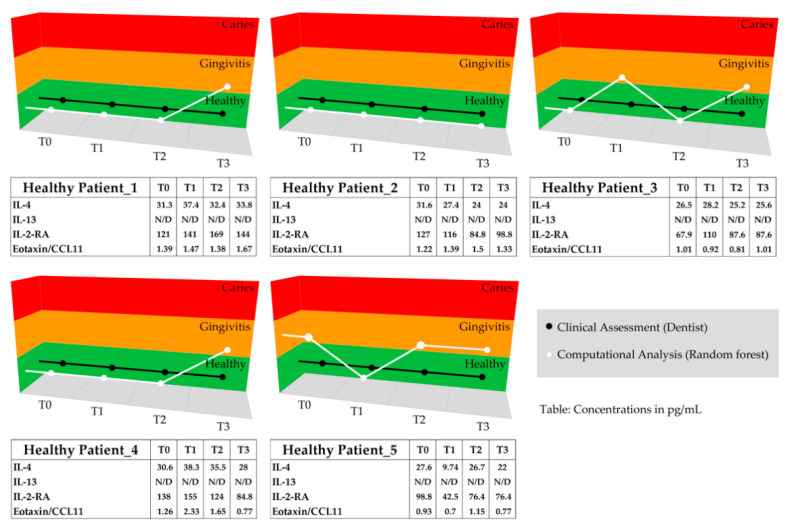

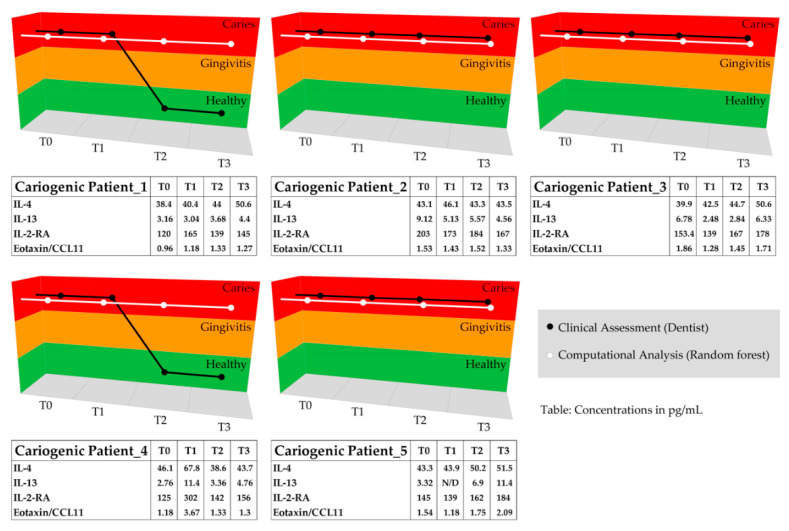
The clinical statuses of five healthy and five caries patients were assessed and classified by dental specialists as well as by biomarker-based RF predictions (Healthy Patient_1 to _5, and Cariogenic Patient_1 to _5). The graphs visualize and compare the classification results based on the RF modeling (white) and the clinical assessment by dental specialists (black) for each timepoint (T0 = baseline, T1 = after 4 months, T2 = after 5 months, and T3 = after 6 months). The tables below the graph show the respective biomarker concentrations in pg/mL, with N/D = values below the detection limit.

RF model prediction was applied on a cohort of 10 subjects (five caries-/gingivitis-free and five with caries) that were part of the repeated measurement group (test dataset). The computational predictions were then compared to clinical classifications performed during the saliva collection appointments by dental practitioners. In addition to timepoint 0 saliva measurements (T0, baseline), three successive measurements were taken four months (T1), five months (T2), and six months (T3) after T0. The comparison of the classifications by the RF model and the clinical classification is visualized in Figure 7. The RF classification of healthy subjects was identical to the clinical classification for 13 of the 20 examination points (Figure 7, Healthy Patients). Minor divergences within the healthy group could be observed in seven instances. The model predicted a condition of gingivitis contrary to to the clinical specialist.

All the caries patients that were followed up during the study changed group during the repeated saliva measurements—from caries to healthy—as indicated by their dental practitioners. The clinical examinations of these patients eventually revealed a caries-free dentition. Changes in diet or self-care plaque control, antibiotic intake, tooth extraction, or professional tooth cleaning were documented and are available in the Supplementary Table S3. Biomarker-based RF prediction for these clinically assessed orally healthy patients generated a different conclusion for four patients (Cariogenic Patient_1 with T2 and T3 Caries, and Cariogenic Patient_4 with T2 and T3 Caries). Based on the RF model and the salivary biomarker levels, these patients remained classified as caries patients (Figure 7). However, overall, there was good agreement, with 16 out of 20 classifications matching between the dental experts and the RF model.

## 4. Discussion

Out of 44 potential biomarkers, a total of four salivary biomarkers were found to exhibit strong potential as classifiers for differentiating between healthy individuals and caries patients. These were the interleukins IL-4 and IL-13, the interleukin receptor IL-2-RA, and the chemokine eotaxin/CCL11. Using, mainly, these four biomarkers, caries patients could be classified into the correct group with a very high degree of certainty (classification error of 2.6%). The RF prediction was based on a training set of 73 subjects and used for the health assessment and prediction of 10 individuals (five caries-/gingivitis-free and five with caries), who were followed over a period of six months (test dataset). The results suggest that the biomarker-based RF prediction of caries patients is more sensitive than clinical assessments by dental specialists. A distinct discrimination between the healthy and gingivitis groups was not possible in this study. Therefore, the initial hypothesis was partially rejected. A clear differentiation of the caries group based on protein but not bacterial biomarkers was enabled and validated with the 10 follow-up subjects.

With respect to IL-4 and IL-13, since 1993, it has been established that the IL-13 gene is closely linked to the IL-4 gene on chromosome 5q 23-31 [61,62], and that there is sequence homology and a shared subunit responsible for signal transduction between these two genes. It has been suggested that both IL-4 and IL-13 are potent in vitro modulators, critical in the regulation of primarily Th2 immune responses [63]. It was shown that Th2 cytokines, such as IL-4, IL-5, and IL-13, together with eotaxin/CCL11, regulate critical aspects of eosinophil recruitment, allergic inflammation, and airway hyperresponsiveness in asthma [62]. A recent study of human monocytes and macrophages confirmed that IL-13 utilizes both the IL-4-RA–JAK2–STAT3 and IL-13-RA–Tyk2–STAT1/STAT6 signaling cascades (JAK = Janus kinase; STAT = signal transducer and activator of transcription proteins), whereas IL-4 can only use the IL-4-RA–JAK1–STAT3/STAT6 axis [64]. A different group investigated the potential of IL-13 as an activator of JAK3–STAT6 signaling in cells expressing IL-2-RG and IL-4-RA [65]. Although initially not stated, Thermo Fisher Scientific confirmed that the utilized “Cytokine 30-Plex Human Panel” assay was developed using the IL-2-RA (Accession Number: P01589) protein. A potential cross-reactivity and unintentional measurement of IL-2-RG in addition to IL-2-RA should be further assessed. The assumption that JAK–STAT signaling could potentially play a role in dental caries was supported by a study on human periodontal ligament cells (HPDLCs). The study revealed that IL-4 is essential in STAT6 activation and the release of the eosinophil-specific chemoattractant eotaxin/CCL11 [66].

Notably, from our results, four of the 20 follow-up measurements in the caries group differed between the clinical assessments by dentists and the computational predictions based on the biomarkers. The clinical examinations revealed a caries-free dentition after placing fillings on the open caries lesions of two patients (Cariogenic Patient_1 and Cariogenic Patient_4 after five and six months). Interestingly, both patients initially showed diverse caries lesions on many teeth simultaneously, meaning that caries treatment was performed on all the open caries lesions. Once they were treated, dentists classified these patients as healthy, according to the study classification criteria. However, molecular analysis indicated the presence of ongoing caries-related immune responses. Persisting biomarkers, which were detected in the saliva of these two multi-caries-treated patients, may have been triggered due to the multiple initial caries lesions and originated from open dentinal tubules or the GCF. Furthermore, the differences in the classifications of healthy patients might be related to the clinical classification protocol utilized during the initial appointments and the enrollment of the study subjects, as the dental examination included screening for caries lesions and their validation by radiographs, which enabled a clear classification. The healthy and gingivitis groups, however, were only characterized by the absence of caries lesions and dental pockets over 3 mm in depth. Bleeding on probing, which is the key parameter for classifying gingivitis patients, was only documented per sextant using PSI scores (gingivitis grouping: no caries lesions, and PSI scores of 1 in ≥4 sextants and ≤2 in ≤2 sextants). This clinical differentiation may have been improved if other bleeding scores had been employed [67].

The low impact of bacterial population levels on the overall classification power of the RF model can potentially be explained by several different aspects. To an extent, periopathogens were not expected to play a role in the differentiation and classification of caries. They were included in this study due to their potential involvement in gingivitis. While no sole bacterial species can be singled out as the causative factor for gingivitis [68], the bacterial species selected for these assays have long been known to be associated with periodontal diseases [69]. Additionally, caries-associated bacteria did not strongly contribute to the overall classification within this study, as shown by the RF modeling. For the qPCR detection, there are two potential causes for the missing values, the absence or small quantity of analyte in the patient saliva or inadequate assay performance. As previously described, all the primer and probe sets were validated using genomic reference DNA using standards ranging in amount from 0.1 pg to 10 ng DNA. The qPCR was repeated on three consecutive days, preparing all the reagents from scratch each time, with the MIQE guidelines being followed throughout the study [70]. The low abundance of *Aggregatibacter actinomycetemcomitans* in the general population has already been reported [71]. However, it appears that the assay performance for *Streptococcus sobrinus* and *Lactobacilli* may have been impaired by the presence of salivary inhibitors. Hence, it cannot be fully excluded that those bacteria are important drivers in the development or identification of dental caries. The total human protease, matrix metalloproteinase, and metallopeptidase inhibitor levels did not enhance the predictability (caries, gingivitis, or healthy) within this study. However, their major contribution to the prediction of caries was not to be expected, as the proteases MMP-8 and MMP-9 are part of a highly complex “protease web”, which is mainly associated with destructive periodontal disease [72].

The lack of research in the field of salivary caries signatures to date might be based on the nature of current caries diagnostics, which are primarily applied with visual examinations including optical caries detection devices, tactile assessments, and radiographs [73,74]. The molecular analyses presented in this publication investigated diverse salivary protein biomarkers and bacterial population levels, and the results could potentially be used to increase the sensitivity of caries detection, as well as to improve caries prevention strategies. A full description of the underlying pathways and involved mechanisms was not possible since this study was first concerned with the screening of potential biomarkers that are able to predict dental caries. The data suggest a potential association between JAK/STAT signaling and linked Th1/Th2 immune responses; however, further research should be conducted in this direction.

Future experiments should also test the universal applicability of the four biomarkers to other patient groups, e.g., patients with periodontitis, heavy smokers, or pregnant and lactating women. Furthermore, potential effects on the oral immune response and microbiome following the consumption of vaporizers and e-cigarettes should be addressed.

## 5. Conclusions

The current research identified four biomarkers (IL-4, IL-13, IL-2-RA, and eotaxin/CCL11) that enabled the correct diagnosis of dental caries in 37 out of 38 patients using RF analysis. Ten subjects were followed over a period of six months, with their oral health status being clinically assessed and compared to biomarker-based RF predictions. We suggest a further validation of the four biomarkers (IL-4, IL-13, IL-2-RA, and eotaxin/CCL11) in the context of JAK/STAT signaling and dental caries. Our results highlight the importance of additional sensitive molecular assays, acting in a way complementary to existing clinical assessment methodologies and enabling a holistic and personalized approach to caries detection and therapy. Biomarker assays may facilitate this approach and allow dentists to accurately keep track of patients’ recovery towards healthy oral microenvironments. This research has laid the foundation for the development of simple and economically feasible saliva-based diagnostic tests aimed at assessing the presence/absence of dental caries in a personalized manner.

## Figures and Tables

**Table 1:** *p*-values of the four biomarkers IL-4, IL-13, IL-2-RA, and eotaxin/CCL11 when comparing patients with caries, patients with gingivitis, and healthy individuals.

Biomarkers	Caries/Gingivitis	Caries/Healthy	Gingivitis/Healthy
IL-4	1.5 × 10^−15^	4.1 × 10^−13^	0.17
IL-13	4.0 × 10^−13^	3.1 × 10^−12^	0.52
IL-2-RA	3.3 × 10^−6^	1.0 × 10^−4^	0.35
Eotaxin/CCL11	8.1 × 10^−5^	4.4 × 10^−4^	0.56

**Table 2:** Confusion matrix comparing random forest (RF) model predictions using biomarkers (top) with the assessments by dental specialists (left).

Status	Healthy	Gingivitis	Caries	Classification Error (%)
Healthy	7	10	1	58.8
Gingivitis	8	7	2	61.1
Caries	1	0	37	2.6

## Data Availability

The data are contained within the article or Supplementary Materials. The data presented in this study are available in Supplementary Data S3.

## Supplementary Materials

The following are available online at https://www.mdpi.com/2075-4426/11/3/235/s1. Table S1: Overview of Cytokine 30-Plex Human Panel, Table S2: Upper and lower detection limits of quantification by assay, Table S3: Compiled dataset including clinical assessment data of all subjects.
